# Building MCP-native hierarchical AI scientist ecosystems: a perspective on scaling multi-agent scientific discovery

**DOI:** 10.3389/frai.2026.1820375

**Published:** 2026-05-13

**Authors:** Ling Yue, Ching-Yun Ko, Pin-Yu Chen, Shimin Di, Shaowu Pan

**Affiliations:** 1 Department of Computer Science, Rensselaer Polytechnic Institute, Troy, NY, United States; 2 IBM Research, Yorktown Heights, NY, United States; 3 School of Computer Science and Engineering, Southeast University, Nanjing, China; 4 Department of Mechanical, Aerospace, and Nuclear Engineering, Rensselaer Polytechnic Institute, Troy, NY, United States

**Keywords:** agent ecosystems, AI for Science, autonomous scientific discovery, Hierarchical coordination, Model Context Protocol, multi-agent systems, organizational design, tool interoperability

## Abstract

Large language models (LLMs) are evolving from chatbots with limited tool-using capabilities to agentic AI systems that can perform deep research, assist in proposing hypotheses, help design experiments, automate data analysis, and draft scientific reports. However, there are currently two bottlenecks limiting LLMs' real-world impact on the broader scientific research community beyond academic demonstrations: *lack of interoperability* (repetitive manual tool-integration is required across scenarios) and *the need for scalable coordination* (unstructured communication and memory become brittle as the number of agents grows). In this Perspective, we argue that the next phase of agentic scientific discovery requires the development of an *ecosystem* of protocol-native agents and tools organized through hierarchies inspired by human society, beyond the current paradigm of a single monolithic “AI scientist”. We use Model Context Protocol (MCP) as a concrete example of an emerging interoperability layer for scientific tool and context exchange, and we propose three complementary pathways to increase the scaling capabilities of an MCP-native scientific ecosystem by addressing the composability issues: (1) MCP servers for high-value scientific tools maintained by domain experts, (2) automated transformation of existing code repositories into MCP services, and (3) autonomous invention and evolution of new agents and workflows. Finally, we provide a practical roadmap for scaling AI-driven scientific discovery by expanding tool supply and coordination in MCP-native scientific ecosystems.

## 1 Introduction

Recent advances in large language models (LLMs) have enabled agents that can interleave reasoning with tool use (Yao et al., 2022; Schick et al., 2023) and collaborate via multi-agent conversation and orchestration frameworks (Wu et al., 2023; Li et al., 2023; Hong et al., 2023). Building on these foundations, a growing body of work explores end-to-end “AI scientist” systems that automate parts of the research lifecycle, including literature review, experimentation, and writing (Lu et al., 2024; Yamada et al., 2025; Gottweis et al., 2025; Schmidgall et al., 2025; Baek et al., 2025; Team et al., 2025; King et al., 2009).

These systems suggest potential to automate and accelerate parts of scientific workflows, but they also expose a practical constraint: high-impact science depends on many tools (simulators, databases, lab instruments, visualization pipelines) and many collaborators (specialists who divide labor and integrate results) (Stokols et al., 2008; National Research Council, 2015; Wilson et al., 2014). As the number of agents increases, each agent begins to resemble a member of a research organization: individually limited in context and attention, but collectively effective when structured well (Simon, 2012).

This Perspective argues that scaling from “single-agent automation” to many-agent scientific organizations requires two ingredients. First, the field needs an interoperability substrate so that tools and capabilities can be composed across models, labs, and domains with minimal glue code (Parnas, 1972; Baldwin and Clark, 2000). Second, as agent populations scale, effective systems will increasingly resemble human research organizations: hierarchical, modular, and grounded in shared artifacts that manage context, accountability, and division of labor (Simon, 2012).

In current systems, both requirements are often unmet (Pan et al., 2025; Wang et al., 2024b). The common failure mode is not that existing systems cannot produce impressive demonstrations, but that their success is brittle outside curated settings (Pan et al., 2025). Tool access is rebuilt inside each host stack, execution environments drift across deployments (Peng, 2011; Sandve et al., 2013; Wilkinson et al., 2016), and multi-agent collaboration relies on unstructured communication (Horling and Lesser, 2004). Our perspective argues that protocols make tools composable, but organizational interfaces make teams composable, and scalable scientific discovery requires both.

The broader protocol landscape has been summarized in Ehtesham et al. (2025). We use the Model Context Protocol (MCP) as a concrete example of an emerging interoperability layer (Anthropic, 2025). Complementary efforts such as Agent2Agent (A2A) address agent-to-agent communication and are positioned as complementing MCP's tool and context interfaces (The Linux Foundation (A2A Protocol Project), 2026). Our organizational claims (hierarchy, shared artifacts, provenance) are largely protocol agnostic; MCP serves to ground the discussion. Concretely, MCP standardizes how LLM hosts connect to external servers that expose tools and resources through a negotiated interface, decoupling fast-moving agent stacks from slower-moving scientific software and infrastructure (Microsoft, 2021; Parnas, 1972; Fielding, 2000). This decoupling is especially important in scientific workflows, where tool execution depends on complex environments, data access policies, and versioned pipelines that are costly to hand-integrate (Wilkinson et al., 2016). We do not propose a new protocol; rather, we argue that protocol-native ecosystems, combined with human-inspired organizational design, are necessary to scale from isolated demonstrations to reusable scientific organizations. By “MCP-native”, we mean that tools and coordination services (e.g., task boards, lab notebooks, provenance stores) are exposed through the protocol interface rather than integrated via host-specific wrappers.

At the same time, interoperability alone does not produce scientific throughput. Many-agent discovery introduces predictable coordination failures: duplicated effort, inconsistent intermediate conclusions, fragmented project state, and weak traceability from claims back to computations and data (Malone and Crowston, 1994; Smith, 1988). Human research groups mitigate these issues through organizational structure and durable shared artifacts (lab notebooks, experiment logs, issue trackers, papers) (Horling and Lesser, 2004; Grosz and Kraus, 1996; Stokols et al., 2008). We argue that MCP-native tool ecosystems and human-inspired organizational design are complementary levers: protocols make tools composable, while organizational interfaces make teams composable.

**Figure 1:**
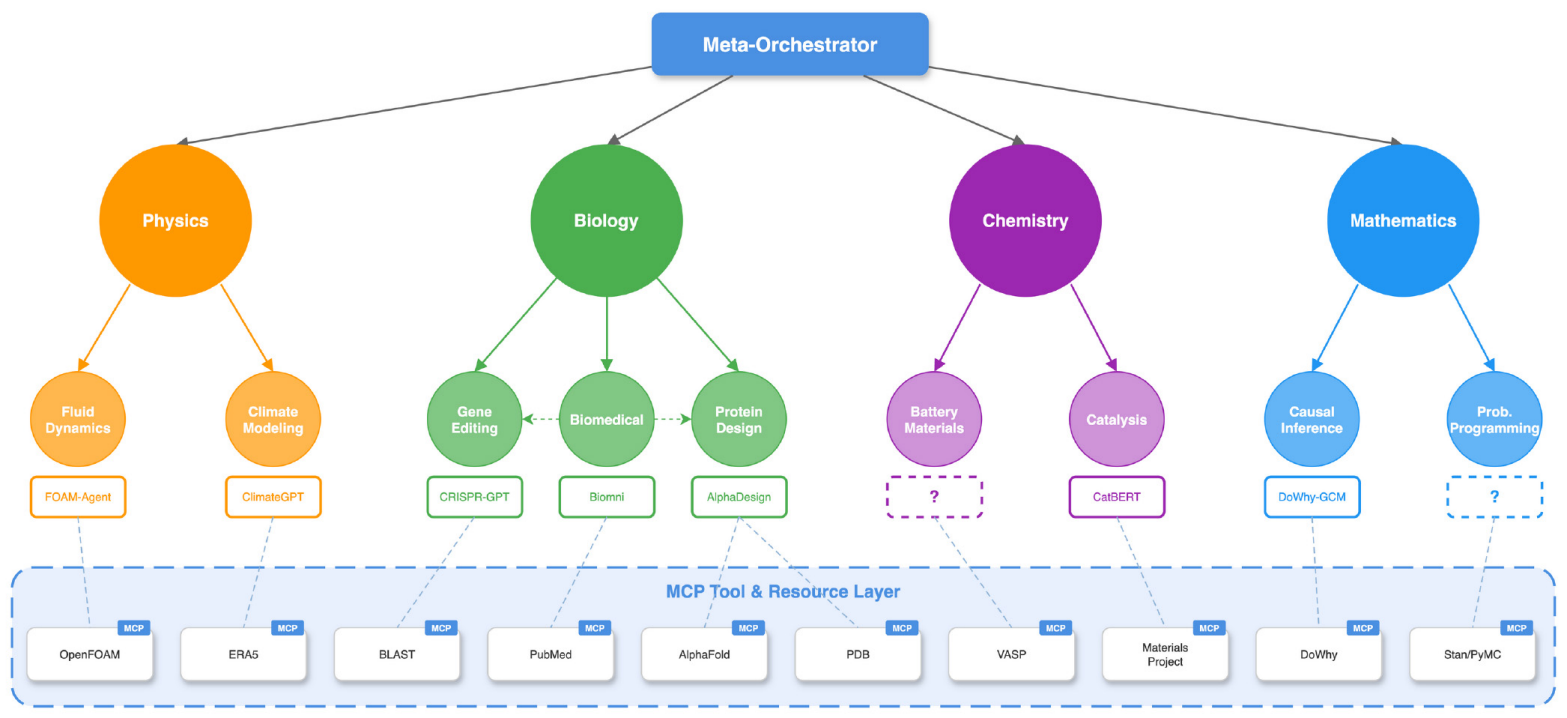
An MCP-native hierarchical AI scientist ecosystem. Each role in the hierarchy is a logical role (not necessarily a single agent) that communicates through information contracts: task specifications and resource budgets propagate downward, while result artifacts and provenance links propagate upward. The three organizational levels are grounded with illustrative scientific domains: a meta-orchestrator (program level) coordinates domain managers for Physics, Biology, Chemistry, and Mathematics (project level), each of which oversees specialist workers such as Fluid Dynamics or Protein Design (task level). Workers access capabilities through domain-specific agent tools (e.g., FOAM-Agent, CRISPR-GPT, AlphaDeep). The bottom layer shows the MCP Tool & Resource Layer, which exposes scientific software and databases (e.g., OpenFOAM, BLAST, AlphaFold, PDB, VASP) as standardized, protocol-accessible services. Solid boxes indicate existing tools; dashed boxes indicate illustrative or emerging capabilities.

Figure 1 provides an overview of this architecture: an organizational hierarchy coordinates multi-domain scientific research through information contracts, while a shared MCP Tool & Resource Layer standardizes access to scientific software and databases, with provenance links connecting high-level claims back to raw tool executions across all layers. The remainder of this Perspective summarizes progress and bottlenecks in “AI scientist” systems, motivates protocol ecosystems with MCP as an example, and then presents an organizational layer and a practical roadmap for growing MCP-native scientific agent ecosystems.

## 2 From standalone agents to AI scientist systems: progress and bottlenecks

Recently, we have witnessed rapid progress in agentic workflows for research (Wang et al., 2024b; Xu et al., 2025). Systems such as *The AI Scientist* and subsequent variants show that LLMs can generate hypotheses, write code, run experiments, and draft papers with minimal human intervention (Lu et al., 2024; Yamada et al., 2025). Complementary efforts emphasize human-in-the-loop and domain grounding, including systems for research ideation (Baek et al., 2025), collaborative “co-scientist” paradigms (Gottweis et al., 2025), and autonomous multi-agent research loops for broader scientific tasks (Schmidgall et al., 2025; Team et al., 2025). Other recent efforts focus on scaling tool access and domain-specific automation: ToolUniverse (Gao et al., 2025) aims to democratize tool access for scientific agents, and BRAD (Pickard et al., 2025) provides an end-to-end multi-agent pipeline for biomarker discovery.

Despite this momentum, most current systems operate as small, fixed teams in curated environments (Shah and White, 2025). This makes demonstrations impressive but difficult to transfer: real scientific work quickly hits the long tail of specialized repositories, heterogeneous runtimes, and evolving datasets (Wilson et al., 2017). For example, extending closed-loop systems like *The AI Scientist* to new domains often requires substantial re-engineering of toolchains, environments, and evaluation harnesses (Lu et al., 2024; Yamada et al., 2025; Kurtzer et al., 2017; Köster and Rahmann, 2012; Di Tommaso et al., 2017; Crusoe et al., 2022). Empirical studies further confirm that reliability and engineering constraints dominate practical agent deployment (Pan et al., 2025). Scaling from “a working demo” to “a reusable scientific organization” is less about prompting and more about engineering reliable interfaces for tool access, shared state, and accountability.

These observations highlight the following two bottlenecks that increasingly limit real-world scientific impacts. (1) The first is tool supply and the *N*×*M* integration problem^1^. Most agent stacks implement per-tool adapters inside the host (Xu et al., 2025). Even when tools are exposed through generic interfaces such as CLIs or web services, contracts for session state, context exchange, and execution traces are often implicit and vary across hosts. As a result, *N* agents accessing *M* tools require *O*(*NM*) *ad-hoc* integrations that are brittle and costly to maintain (Parnas, 1972; Ehtesham et al., 2025). Interoperability protocols aim to replace host-specific glue code with reusable services under explicit, negotiated contracts. MCP is a concrete instance: it standardizes how agent hosts connect to external tool/context servers, providing capability discovery, context exchange, and auditable traces as shared infrastructure (Anthropic, 2025). In scientific settings, this burden is amplified by environment complexity (compiled dependencies, GPU/HPC constraints, container images) and data governance (dataset snapshots, access control, provenance), and “running the tool” is often inseparable from reproducing its execution context (Wilkinson et al., 2016; Sandve et al., 2013; Kurtzer et al., 2017). Silent behavioral drift across tool versions can further invalidate downstream conclusions (Guo et al., 2024). (2) The second bottleneck is coordination and context management at scale. As the number of agents grows, flat communication patterns become costly and brittle because the number of potential peer-to-peer links grows quadratically (Malone and Crowston, 1994; Conway, 1968). Moreover, LLM agents have bounded context windows and imperfect long-horizon memory, making it difficult for any single agent to hold the full state of a complex research program. Scientific work also raises the bar for coordination quality: teams must track experimental conditions, manage uncertainty, and resolve disagreements through replication, ablations, or alternative methods (Horling and Lesser, 2004). Without durable shared artifacts and explicit traceability from claims to evidence, multi-agent systems can produce plausible narratives that are difficult to audit, reproduce, or build upon (Park et al., 2023).

These bottlenecks motivate the two design directions developed in the rest of this Perspective: protocol ecosystems (with MCP as a concrete instance) to reduce integration debt and grow tool supply, and a human-inspired organizational layer to shape information flow, externalize memory into shared artifacts, and preserve scientific-grade accountability as agent populations scale.

## 3 Why protocol ecosystems matter: MCP as an interoperability layer

We argue that “agentic science” should be built on open, protocol-based ecosystems rather than bespoke, closed integrations (Fielding, 2000; Parnas, 1972). MCP is one prominent example: it standardizes how hosts (LLM applications) connect to servers that provide resources, prompts, and tools, using JSON-RPC 2.0 over stateful connections with capability negotiation. MCP is explicitly inspired by the Language Server Protocol (LSP), which enabled a large ecosystem of reusable language tooling across editors via a common protocol (Anthropic, 2025; Microsoft, 2021).

From a scientific discovery perspective, protocol ecosystems offer four benefits. First, they enable composable tool access with clear boundaries. Protocol-defined tools can be versioned, tested, and sandboxed independently of the agents that call them, which decouples fast-moving agent stacks from slower-moving scientific software and infrastructure. Second, they treat context as a first-class interface. Scientific work requires passing rich context (hypotheses, experimental settings, data provenance, intermediate conclusions), and MCP's resources and stateful sessions support structured context exchange beyond prompt-only interfaces (Anthropic, 2025). Third, they lower the cost of reproducibility and provenance (Belhajjame et al., 2013). When tools are exposed through standardized interfaces, we can more systematically log tool calls, inputs/outputs, and environment versions. Protocols do not guarantee rigor on their own, but they make it easier to build rigorous, auditable pipelines (Wilkinson et al., 2016). Finally, a protocol is only as useful as the ecosystem of servers that implement it. We therefore emphasize the “tool supply” problem as a first-class research and engineering challenge (Huang and Lapp, 2013; Wilson et al., 2017). For example, Code2MCP (Ouyang et al., 2025) and ToolRosetta (Di et al., 2026) provide automated pathways to populate protocol ecosystems by converting existing code repositories into deployable, standardized tool services with minimal human intervention. Such agentic conversion methods hold great potential to unlock the long tail of domain software required for scalable scientific discovery. Proprietary tools lacking native MCP interfaces can be wrapped behind gateway servers that handle authentication at the boundary (e.g., via OAuth 2.0 or API-key vaulting), enforcing access-control policies and usage auditing while keeping raw credentials out of agent context.

## 4 Scaling many-agent discovery needs human-inspired organizational design

MCP-like interoperability reduces the *N*×*M* integration burden, but does not by itself make large agent populations effective. As agent count grows, the dominant bottleneck shifts to coordination (Malone and Crowston, 1994). Human research groups mitigate this through organizational structure and shared artifacts (National Research Council, 2015; Horling and Lesser, 2004); scalable many-agent discovery similarly requires an explicit organizational layer for task allocation, shared state, conflict resolution, and accountability.

### 4.1 What breaks when we scale beyond small agent teams

When coordination is left implicit, large agent teams fail in predictable ways. Agents unknowingly duplicate subtasks and produce inconsistent partial results (Malone and Crowston, 1994). Project state fragments across private chat histories, causing assumptions to drift and eroding shared ground truth. Communication scales poorly in flat peer-to-peer structures: the number of possible interactions grows quadratically, diluting high-signal updates with noise (Horling and Lesser, 2004). Unresolved conflicts accumulate without mechanisms to detect and adjudicate disagreements. Finally, without structured provenance, claims become untraceable to specific tool executions, data versions, and experimental settings. These breakdowns are structural consequences of scaling bounded-context agents (Park et al., 2023; Wang et al., 2023).

### 4.2 Organizational primitives as first-class interfaces

To counter these failures, we advocate an artifact-centered organizational layer built from a small set of primitives that can be standardized and reused across domains:

- A task interface (assignment, ownership, and budgets). Every subtask should have an explicit owner, inputs, success criteria, and resource limits. This supports de-duplication, progress tracking, and controlled exploration (Smith, 1988; Malone and Crowston, 1994).
- A shared artifact interface (externalized state). Hypotheses, datasets, experiment configurations, results, and summaries should live as versioned, inspectable artifacts rather than private chat context. These artifacts become the durable “memory” of the organization (Simon, 2012; Park et al., 2023).
- A decision and disagreement interface (escalation and repair). When results conflict, the system should create structured disagreement records and trigger repair actions (replication, ablations, alternative methods, or human review), reducing silent divergence (Grosz and Kraus, 1996; Tambe, 1997). Multi-agent debate (Du et al., 2024) provides one natural implementation strategy for such structured argumentation within agent teams.
- A provenance interface (traceability). Claims should link to evidence artifacts and execution traces (tool calls, environment versions, data lineage), supporting audit and reproducibility (Wilkinson et al., 2016; Groth and Moreau, 2013). A key design principle is that higher-level summaries should be *compressions with evidence pointers*, not replacements for evidence: every summary that propagates upward through the hierarchy retains structured links to the underlying task records, result artifacts, execution traces, data snapshots, and environment or version information, so that any claim can be audited back to raw computations and data.

In a MCP-native ecosystem, these primitives can be implemented as protocol-accessible servers and resources (e.g., task boards, lab notebooks, provenance stores), making coordination logic modular and composable across labs and agent frameworks.

### 4.3 A minimal hierarchy is an information contract, not just a role chart

Figure 1 illustrates a pragmatic hierarchy that functions primarily as an information contract rather than a static role chart. By “information contract” we mean both a conceptual description of what information should move between roles and, operationally, a concrete structured schema (e.g., typed task records, result bundles, decision records, and provenance logs) that can be enforced and validated at each layer boundary. Three layers serve as a minimal reference; actual deployments may be hierarchical, hybrid, or dynamically reconfigured (Horling and Lesser, 2004). Each role in the hierarchy, including the meta-orchestrator, is a *logical role*, not necessarily a single LLM agent; it may be instantiated by one agent, a committee of agents with shared context, or a recursively organized coordination unit. What defines a role is the information contract it fulfills (what it receives, what it produces, and what it is accountable for), not the number of agents behind it. At the task level, specialist workers execute concrete actions via MCP tools and write back result artifacts together with execution traces (tool calls, inputs/outputs, software versions, random seeds, and environment snapshots). At the project level, domain managers allocate tasks, maintain shared project context through curated artifacts, reconcile conflicts, and distill evidence into structured summaries and decision records, each retaining links to the underlying task-level traces. At the program level, meta-orchestrators define long-horizon objectives, allocate budgets across projects, and integrate project-level summaries into a portfolio view of progress and uncertainty. For example, a program-level claim such as “Method A outperforms Method B on dataset X” links to a project-level decision record containing the comparison protocol, which in turn links to individual task artifacts with the exact tool calls, data versions, and output checksums. This cross-layer provenance chain ensures that any high-level finding can be audited back to raw data and tool-execution logs.

Importantly, the hierarchy constrains *information flow*, not *decision-making autonomy* (Simon, 2012): agents retain full freedom to choose methods and tools within each layer, while the hierarchy governs what summaries propagate upward and what specifications flow downward. For tasks requiring decentralized exploration (e.g., early-stage hypothesis generation), the manager layer can relax into a peer-to-peer or blackboard-style mode (Nii, 1986), re-engaging hierarchy when results must be reconciled. Most information exchange remains local, with only compressed, decision-relevant updates propagating upward and long-horizon state externalized through shared artifacts, reducing dependence on any single agent's context window. In this sense, hierarchy scales not by increasing conversation volume but by shaping information flow, mirroring how human laboratories grow through specialization combined with explicit interfaces for tasks, artifacts, and accountability (Conway, 1968).

## 5 Roadmap: three pathways to grow an MCP-native scientific agent ecosystem

**Figure 2:**
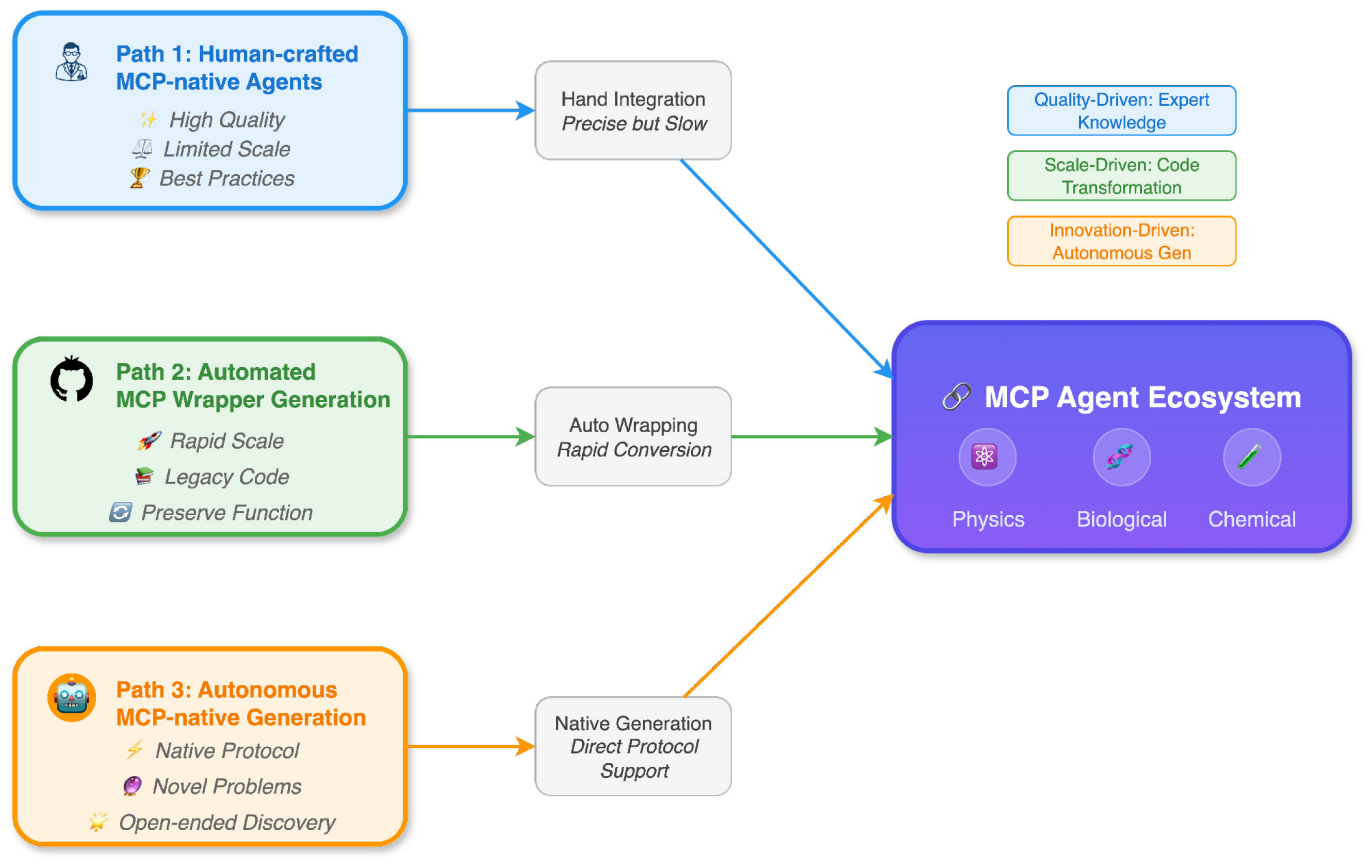
Three pathways to build an MCP agent ecosystem. Human-crafted MCP-native agents (Pathway 1) provide high-quality foundations, automated MCP wrapper generation (Pathway 2) enables rapid scaling from legacy code, and autonomous MCP-native generation (Pathway 3) creates novel agents for unprecedented problems. All three paths converge into a unified MCP agent ecosystem spanning diverse scientific domains.

To make this vision actionable, we outline three complementary pathways for bootstrapping and scaling an MCP-native ecosystem (Figure 2).

Pathway 1: expert-crafted MCP servers for high-value scientific tools. In the short term, the highest-leverage approach is to build MCP servers for widely used scientific tools (e.g., simulation packages, lab automation APIs, scientific databases). Human experts can encode domain constraints, safety checks, and best practices. These “gold standard” servers can also serve as reference implementations for evaluating automated conversion methods (Crusoe et al., 2022; Di Tommaso et al., 2017; Kurtzer et al., 2017).

Pathway 2: automated code-to-MCP transformation to unlock the long tail. Most scientific capabilities live in open-source repositories that were never designed as agent-ready services (Kurtzer et al., 2017). Automated frameworks such as Code2MCP (Ouyang et al., 2025) and ToolRosetta (Di et al., 2026) propose converting arbitrary repositories into standardized tool services through multi-agent workflows that analyze code, reproduce environments, design tool schemas, and iteratively debug via self-correction loops. We view this pathway as essential for scaling tool supply and enabling scientific agents to access specialized methods without extensive hand engineering (Guo et al., 2024).

Pathway 3: autonomous invention and evolution of new agents and workflows. In the longer term, the ecosystem should not only wrap existing tools but also invent new ones. Recent work on automated agent design and self-improving systems, including Automated Design of Agentic Systems (ADAS), AFlow, and the Darwin Gödel Machine, suggests that agents can search over workflow code and iteratively improve themselves using execution feedback (Hu et al., 2024; Zhang et al., 2024, 2025). A recent survey of workflow optimization for LLM agents (Yue et al., 2026) further highlights the trend from static, template-based pipelines toward dynamic runtime graphs that can be composed and adapted on the fly. In an MCP-native setting, new agents can be materialized as new MCP servers (tools) or as new orchestration policies (managers), expanding the ecosystem over time (Shinn et al., 2023).

## 6 Discussion

Our perspective reframes “AI scientist” progress as an ecosystem problem. Once protocol layers like MCP reduce the *N*×*M* integration burden between agents and tools, the main constraints become whether tool supply can scale with scientific-grade reliability and whether large agent populations can coordinate through organizational structure and shared artifacts.

Several recent efforts address complementary aspects: ToolUniverse (Gao et al., 2025) democratizes tool access (complementary to our Pathways 1–2 but without organizational coordination); Mixture-of-Agents (Wang et al., 2024a) boosts capability through flat ensembles (our framework adds hierarchy, artifacts, and provenance for auditable scaling); BRAD (Pickard et al., 2025) provides a domain-specific pipeline for biomarker discovery (illustrating workflows exposable as MCP services); and multi-agent debate (Du et al., 2024) offers structured argumentation aligned with our disagreement interface. What distinguishes our perspective is the argument that protocol ecosystems and organizational design are jointly necessary.

Our emphasis on organizational structure reflects the stronger requirements of scientific discovery (Ioannidis, 2005). The cost of error is highly asymmetric: a false positive can waste days of compute or weeks of lab time, and a silently wrong result can contaminate downstream hypotheses (Munafò et al., 2017). Conclusions also hinge on software versions, random seeds, data snapshots, and experimental settings (Wilkinson et al., 2016; Sandve et al., 2013). As a result, “doing the task” is inseparable from “being able to rerun and audit the task”, which is why we emphasize artifact-centered coordination and tool interfaces as contracts that make evidence traceable.

In the near term, Pathway 2 is strategically important but still brittle in practice: conversion pipelines can struggle to reproduce environments, infer correct schemas, and detect silent wrong outputs (Ouyang et al., 2025). This direction arguably receives less attention than its leverage warrants. Advancing it requires treating conversion as a testing-and-provenance problem, with standardized harnesses for environment capture, regression checks, and versioned tool contracts (Guo et al., 2024), defaulting to conservative behaviors such as version reporting, structured logging, and verification hooks.

Over the longer term, Pathway 3 raises a deeper question: what should automatically generated scientific workflows look like? A key risk is that self-improving systems optimize for short-horizon signals while accumulating hidden technical debt. Useful self-evolution must therefore be coupled with auditability and rollback, preserving reproducibility and evidence attribution even as orchestration policies and tool libraries evolve.

Progress will likely depend on ecosystem-level evaluations that go beyond task success rates (Liu et al., 2023). Practical metrics include tool coverage (how much of a domain toolchain is accessible), integration cost (time and maintenance burden per tool), coordination cost (redundant work and conflict rates as teams scale), and reproducibility under continuous evolution (the fraction of results that remain rerunnable as tools, environments, and agents change). These evaluation directions align with the core thesis of this Perspective: protocols make tools composable, but organizational design makes teams composable, and both are required for scalable scientific discovery.

## Data Availability

The original contributions presented in the study are included in the article/supplementary material, further inquiries can be directed to the corresponding author.

## Footnotes

1 *N* refers to the number of models/agents, and *M* refers to the number of tools.
